# Hexa-μ-chlorido-hexa­chlorido(η^6^-hexa­methyl­benzene)trialuminium(III)lanthanum(III) benzene solvate

**DOI:** 10.1107/S1600536809004899

**Published:** 2009-02-18

**Authors:** Alexander S. Filatov, Sarah N. Gifford, D. Krishna Kumar, Marina A. Petrukhina

**Affiliations:** a Department of Chemistry, University at Albany, 1400 Washington Avenue, Albany, NY 12222, USA

## Abstract

In the title compound, [Al_3_LaCl_12_(C_12_H_18_)]·C_6_H_6_, all mol­ecules are located on a mirror plane. Three chloridoaluminate groups and a hexa­methyl­benzene mol­ecule are bound to the central lanthanum(III) ion, forming a distorted penta­gonal bipyramid with the η^6^-coordinated arene located at the apical position. The hexa­methyl­benzene ligand disordered between two orientations in a 1:1 ratio is also involved in parallel-slipped π–π stacking inter­molecular inter­actions with a benzene solvent mol­ecule [centroid–centroid distance 3.612 (4) Å].

## Related literature

For the previously characterized lanthanum chloro­aluminate and chloro­gallate complexes, see: Filatov *et al.* (2008 ▶). For a recent review of other lanthanide chloro­aluminate complexes, see: Bochkarev (2002 ▶). For complexes of lanthanide chloro­gallates with polycyclic aromatic systems, see: Gorlov *et al*. (2008 ▶).
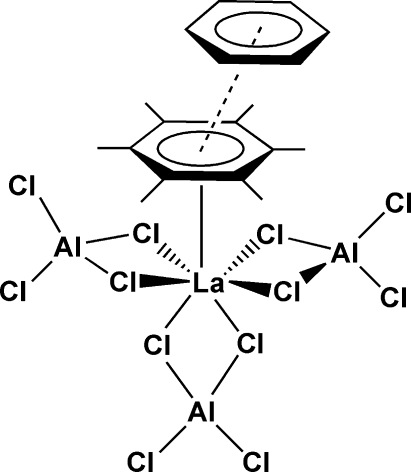


## Experimental

### 

#### Crystal data

[Al_3_LaCl_12_(C_12_H_18_)]·C_6_H_6_
*M* *_r_* = 885.62
Orthorhombic, 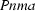

*a* = 12.2127 (6) Å
*b* = 16.4205 (8) Å
*c* = 16.9790 (8) Å
*V* = 3404.9 (3) Å^3^
*Z* = 4
Mo- *K*α radiation
μ = 2.28 mm^−1^
*T* = 173 K
0.22 × 0.20 × 0.16 mm

#### Data collection

Bruker SMART APEX CCD area-detector diffractometer
Absorption correction: multi-scan (*SADABS*; Bruker, 2003 ▶) *T*_min_ = 0.613, *T*_max_ = 0.697
28535 measured reflections
4250 independent reflections
3911 reflections with *I* > 2σ(*I*)
*R*_int_ = 0.018

#### Refinement

*R*[*F* ^2^ > 2σ(*F* ^2^)] = 0.025
*wR*(*F* ^2^) = 0.063
*S* = 1.05
4250 reflections
189 parameters
H-atom parameters not refined
Δρ_max_ = 0.89 e Å^−3^
Δρ_min_ = −0.99 e Å^−3^

### 

Data collection: *SMART* (Bruker, 2003 ▶); cell refinement: *SAINT* (Bruker, 2003 ▶); data reduction: *SAINT*; program(s) used to solve structure: *SHELXS97* (Sheldrick, 2008 ▶); program(s) used to refine structure: *SHELXL97* (Sheldrick, 2008 ▶); molecular graphics: *SHELXTL* (Sheldrick, 2008 ▶) and *ORTEP-3 for Windows* (Farrugia, 1997 ▶); software used to prepare material for publication: *SHELXTL* and *publCIF* (Westrip, 2009 ▶).

## Supplementary Material

Crystal structure: contains datablocks global, I. DOI: 10.1107/S1600536809004899/cv2516sup1.cif

Structure factors: contains datablocks I. DOI: 10.1107/S1600536809004899/cv2516Isup2.hkl

Additional supplementary materials:  crystallographic information; 3D view; checkCIF report

## Figures and Tables

**Table 1:** Selected bond lengths (Å)

La1—C1	2.945 (3)
La1—C2	2.965 (2)
La1—C3	2.957 (3)
La1—C4	2.941 (4)
La1—Cl3	2.9128 (5)
La1—Cl4	2.9298 (6)
La1—Cl5	2.9083 (7)
La1—Cl6	2.9097 (7)
La1—*Cg*1	2.613 (3)

**Fig. 1.**
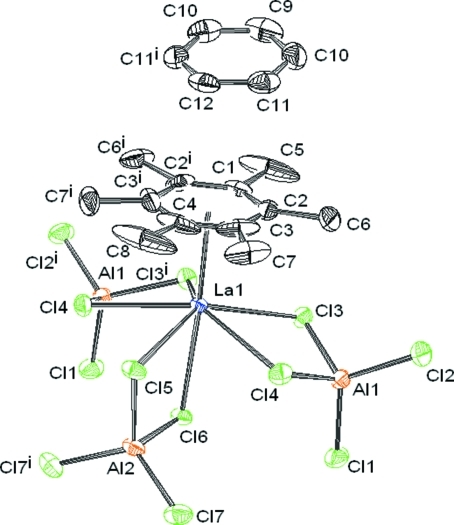
A view of the molecular structure of (I), along with the atom numbering scheme [symmetry code: (i) x, -y + 1/2, z]. Displacement ellipsoids are drawn at the 30% probability level. Hydrogen atoms and disordered parts are omitted for clarity.

**Fig. 2.**
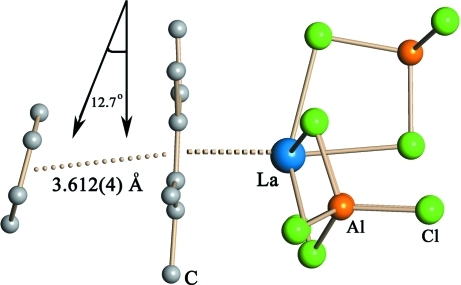
A view of the molecular structure of (I) showing π-π stacking interactions between coordinated C6Me6 and benzene rings.

## supplementary crystallographic information

### Comment

We have recently reported X-ray structural characterization of the first two lanthanum(III) chloroaluminate complexes with neutral arenes, [La(*η*^6^-C_6_H_5_Me)(AlCl_4_)_3_] and [La(*η*^6^-C_6_Me_6_)(AlCl_4_)_3_], as well as of the first lanthanum(III) chlorogallate complex, [La(*η*^6^-C_6_Me_6_)(GaCl_4_)_3_] (Filatov *et al.*, 2008). The [La(*η*^6^-C_6_Me_6_)(AlCl_4_)_3_]^.^0.5C_6_H_6_ complex crystallizes in the monoclinic *P*2_1_/*c* space group with the *β* angle being close to 90° (*β* = 90.27°). We later found that under slightly different experimental conditions, namely at a higher temperature (285 *versus* 273 K), the lanthanum complex with hexamethylbenzene, [La(*η*^6^-C_6_Me_6_)(AlCl_4_)_3_]^.^C_6_H_6_ (I), is crystallized.

The molecular structure of (I) is comprised of the three chloroaluminate groups and a hexamethylbenzene molecule bound to the central lanthanum(III) ion (Fig.1). The coordination polyhedron is a distorted pentagonal bipyramid with the *η*^6^-arene located at the apical position. The La–C bond distances span from 2.941 (4) to 2.965 (2) Å with a La–centroid distance being 2.613 (3) Å. These distances are comparable to those found in the previously reported complex [La(*η*^6^-C_6_Me_6_)(AlCl_4_)_3_]^.^0.5C_6_H_6_  (II) [La—C 2.927 (7)–3.035 (7)Å; La–centroid 2.633 (7)Å].

In (I), coordinated hexamethylbenzene is engaged into π-π stacking interactions with a solvent benzene molecule. The intercentroid distance between their 6-membered rings is 3.612 (4) Å. The two ring planes are not parallel and the dihedral angle is 12.7° (Fig.2). In the above hemisolvate (II), on the contrary, both benzene faces are involved in π-π stacking interactions as benzene is sandwiched between two hexamethylbenzene molecules. The distance between the centroids of the hexamethylbenzene and benzene rings (3.688 (4) Å) is noticeably longer than that found in (I).

### Experimental

LaCl_3_ (100 mg, 0.41 mmol), AlCl_3_ (163 mg, 1.22 mmol), hexamethylbenzene (66 mg, 0.41 mmol) and an excess of aluminium foil were placed into a Schlenk flask inside the glove box. Benzene (10 ml) was added to the flask and the mixture was refluxed for two hours. The LaCl_3_, AlCl_3_, and hexamethylbenzene dissolved completely to give a yellow solution. The solution was filtered while hot through a pad of Celite and then kept at 12°C under argon for 2 days to afford a yellow crystalline material. Yield: 240 mg (65%). IR data (cm^-1^): 3091 (w), 3071 (w), 3036 (w), 1598 (*m*), 1531 (w), 1478 (*m*), 1423 (*s*), 1382 (*m*), 1332 (*m*), 1272 (*m*), 1180 (w), 1076 (w), 983 (w), 824 (w), 677 (*s*).

### Refinement

All C—H atoms were refined using the riding model approximation, with C—H = 0.95–0.98Å [*U*_iso_(H) = 1.2 or 1.5Ueq(C)]. All other atoms were refined anisotropically. Large anisotropy of the carbon atoms of hexamethylbenzene suggests the presence of disorder. It was modeled over two rotational orientations in a 1:1 ratio. The C5 and C8 carbon atoms lie on a mirror plane and are constrained to have identical anisotropic displacement parameters (EADP command in the *SHELXL* realm).

### Crystal data

**Table:**

[Al_3_LaCl_12_(C_12_H_18_)]·C_6_H_6_	*F*(000) = 1728
*M**_r_* = 885.62	*D*_x_ = 1.728 Mg m^−^^3^
Orthorhombic, *P**n**m**a*	Mo *K*α radiation, λ = 0.71073 Å
Hall symbol: -P 2ac 2n	Cell parameters from 7019 reflections
*a* = 12.2127 (6) Å	θ = 2.5–28.2°
*b* = 16.4205 (8) Å	µ = 2.28 mm^−^^1^
*c* = 16.9790 (8) Å	*T* = 173 K
*V* = 3404.9 (3) Å^3^	Block, yellow
*Z* = 4	0.22 × 0.20 × 0.16 mm

### Data collection

**Table:**

Bruker SMART APEX CCD area-detector diffractometer	4250 independent reflections
Radiation source: fine-focus sealed tube	3911 reflections with *I* > 2σ(*I*)
graphite	*R*_int_ = 0.018
0.30° ω scans	θ_max_ = 28.3°, θ_min_ = 2.1°
Absorption correction: multi-scan (*SADABS*; Bruker, 2003)	*h* = −15→16
*T*_min_ = 0.613, *T*_max_ = 0.697	*k* = −21→21
28535 measured reflections	*l* = −22→22

### Refinement

**Table:**

Refinement on *F*^2^	Primary atom site location: structure-invariant direct methods
Least-squares matrix: full	Secondary atom site location: difference Fourier map
*R*[*F*^2^ > 2σ(*F*^2^)] = 0.025	Hydrogen site location: inferred from neighbouring sites
*wR*(*F*^2^) = 0.063	H-atom parameters not refined
*S* = 1.05	*w* = 1/[σ^2^(*F*_o_^2^) + (0.0304*P*)^2^ + 3.1621*P*] where *P* = (*F*_o_^2^ + 2*F*_c_^2^)/3
4250 reflections	(Δ/σ)_max_ < 0.001
189 parameters	Δρ_max_ = 0.89 e Å^−^^3^
0 restraints	Δρ_min_ = −0.98 e Å^−^^3^

### Special details

**Table:**

Geometry. All e.s.d.'s (except the e.s.d. in the dihedral angle between two l.s. planes) are estimated using the full covariance matrix. The cell e.s.d.'s are taken into account individually in the estimation of e.s.d.'s in distances, angles and torsion angles; correlations between e.s.d.'s in cell parameters are only used when they are defined by crystal symmetry. An approximate (isotropic) treatment of cell e.s.d.'s is used for estimating e.s.d.'s involving l.s. planes.
Refinement. Refinement of *F*^2^ against ALL reflections. The weighted *R*-factor *wR* and goodness of fit *S* are based on *F*^2^, conventional *R*-factors *R* are based on *F*, with *F* set to zero for negative *F*^2^. The threshold expression of *F*^2^ > σ(*F*^2^) is used only for calculating *R*-factors(gt) *etc*. and is not relevant to the choice of reflections for refinement. *R*-factors based on *F*^2^ are statistically about twice as large as those based on *F*, and *R*- factors based on ALL data will be even larger.

### Fractional atomic coordinates and isotropic or equivalent isotropic displacement parameters (Å^2^)

**Table:**

	*x*	*y*	*z*	*U*_iso_*/*U*_eq_	Occ. (<1)
La1	0.106393 (12)	0.2500	0.425620 (9)	0.02758 (6)	
Al1	0.09537 (6)	0.04272 (4)	0.32343 (5)	0.04146 (16)	
Al2	−0.20200 (8)	0.2500	0.41914 (6)	0.0403 (2)	
Cl1	−0.02765 (6)	0.03298 (4)	0.23836 (5)	0.06224 (19)	
Cl2	0.18966 (7)	−0.06204 (4)	0.34109 (6)	0.0684 (2)	
Cl3	0.19872 (5)	0.14875 (3)	0.30139 (3)	0.04206 (13)	
Cl4	0.02586 (5)	0.08226 (4)	0.43571 (3)	0.04489 (14)	
Cl5	−0.09121 (6)	0.2500	0.52121 (4)	0.04250 (18)	
Cl6	−0.08346 (6)	0.2500	0.32208 (4)	0.03682 (16)	
Cl7	−0.29273 (6)	0.14181 (5)	0.41927 (4)	0.05841 (18)	
C1	0.3384 (3)	0.2500	0.4729 (2)	0.0515 (10)	
C2	0.3003 (3)	0.17691 (17)	0.50333 (19)	0.0609 (8)	
C3	0.2212 (3)	0.1777 (3)	0.5621 (2)	0.0823 (13)	
C4	0.1820 (3)	0.2500	0.5901 (2)	0.096 (3)	
C5	0.4271 (4)	0.2500	0.4107 (3)	0.194 (5)	
H5A	0.4789	0.2943	0.4214	0.291*	0.50
H5B	0.3940	0.2580	0.3587	0.291*	0.50
H5C	0.4659	0.1978	0.4118	0.291*	0.50
C6	0.3258 (8)	0.0879 (6)	0.4928 (7)	0.087 (3)	0.50
H6A	0.3781	0.0706	0.5332	0.130*	0.50
H6B	0.3577	0.0791	0.4405	0.130*	0.50
H6C	0.2583	0.0561	0.4977	0.130*	0.50
C7	0.1582 (8)	0.1206 (6)	0.6166 (5)	0.093 (3)	0.50
H7A	0.1812	0.1299	0.6711	0.139*	0.50
H7B	0.1735	0.0640	0.6019	0.139*	0.50
H7C	0.0796	0.1312	0.6117	0.139*	0.50
C6X	0.3773 (8)	0.1077 (7)	0.4617 (6)	0.084 (4)	0.50
H6X1	0.4398	0.0956	0.4959	0.126*	0.50
H6X2	0.4039	0.1281	0.4109	0.126*	0.50
H6X3	0.3344	0.0581	0.4533	0.126*	0.50
C7X	0.2146 (9)	0.0787 (5)	0.5824 (6)	0.092 (3)	0.50
H7X1	0.1425	0.0659	0.6045	0.137*	0.50
H7X2	0.2716	0.0646	0.6206	0.137*	0.50
H7X3	0.2257	0.0473	0.5339	0.137*	0.50
C8	0.1026 (4)	0.2500	0.6586 (3)	0.194 (5)	
H8A	0.0691	0.1960	0.6636	0.291*	0.50
H8B	0.0454	0.2907	0.6494	0.291*	0.50
H8C	0.1420	0.2633	0.7072	0.291*	0.50
C9	0.5948 (4)	0.2500	0.6119 (3)	0.088 (2)	
H9	0.6600	0.2500	0.5814	0.106*	
C10	0.5464 (3)	0.1771 (2)	0.6345 (2)	0.0750 (10)	
H10	0.5779	0.1265	0.6193	0.090*	
C11	0.4547 (3)	0.1790 (2)	0.6780 (2)	0.0679 (9)	
H11	0.4212	0.1293	0.6936	0.082*	
C12	0.4094 (3)	0.2500	0.7000 (3)	0.0646 (12)	
H12	0.3449	0.2500	0.7312	0.077*	

### Atomic displacement parameters (Å^2^)

**Table:**

	*U*^11^	*U*^22^	*U*^33^	*U*^12^	*U*^13^	*U*^23^
La1	0.02175 (8)	0.03570 (10)	0.02528 (9)	0.000	0.00028 (5)	0.000
Al1	0.0428 (4)	0.0342 (3)	0.0475 (4)	−0.0003 (3)	−0.0093 (3)	−0.0026 (3)
Al2	0.0247 (4)	0.0577 (6)	0.0384 (5)	0.000	0.0008 (4)	0.000
Cl1	0.0664 (4)	0.0525 (4)	0.0678 (4)	0.0015 (3)	−0.0311 (3)	−0.0082 (3)
Cl2	0.0666 (4)	0.0438 (3)	0.0947 (6)	0.0136 (3)	−0.0232 (4)	−0.0036 (4)
Cl3	0.0434 (3)	0.0424 (3)	0.0404 (3)	−0.0030 (2)	0.0099 (2)	−0.0068 (2)
Cl4	0.0460 (3)	0.0431 (3)	0.0456 (3)	−0.0101 (2)	0.0013 (2)	0.0080 (2)
Cl5	0.0288 (3)	0.0687 (5)	0.0300 (3)	0.000	0.0031 (3)	0.000
Cl6	0.0277 (3)	0.0530 (4)	0.0298 (3)	0.000	−0.0025 (3)	0.000
Cl7	0.0418 (3)	0.0720 (5)	0.0615 (4)	−0.0171 (3)	0.0028 (3)	−0.0021 (3)
C1	0.0227 (14)	0.099 (3)	0.0325 (16)	0.000	−0.0035 (12)	0.000
C2	0.0654 (17)	0.0486 (14)	0.0688 (18)	0.0173 (13)	−0.0438 (16)	−0.0151 (13)
C3	0.071 (2)	0.114 (3)	0.0617 (19)	−0.055 (2)	−0.0416 (18)	0.055 (2)
C4	0.0269 (19)	0.237 (9)	0.0259 (18)	0.000	−0.0009 (14)	0.000
C5	0.0328 (17)	0.507 (15)	0.0414 (19)	0.000	0.0080 (14)	0.000
C6	0.082 (7)	0.060 (5)	0.118 (9)	0.034 (5)	−0.054 (6)	−0.025 (5)
C7	0.099 (7)	0.110 (7)	0.070 (5)	−0.061 (6)	−0.042 (5)	0.058 (5)
C6X	0.075 (6)	0.089 (8)	0.087 (7)	0.044 (6)	−0.037 (5)	−0.040 (6)
C7X	0.111 (8)	0.067 (5)	0.097 (7)	−0.032 (5)	−0.053 (6)	0.048 (5)
C8	0.0328 (17)	0.507 (15)	0.0414 (19)	0.000	0.0080 (14)	0.000
C9	0.052 (3)	0.172 (7)	0.041 (2)	0.000	0.0042 (19)	0.000
C10	0.081 (2)	0.080 (2)	0.0634 (19)	0.029 (2)	−0.0217 (18)	−0.0169 (17)
C11	0.070 (2)	0.069 (2)	0.0653 (19)	−0.0142 (17)	−0.0277 (16)	0.0189 (16)
C12	0.039 (2)	0.105 (4)	0.049 (2)	0.000	−0.0136 (17)	0.000

### Geometric parameters (Å, °)

**Table:**

La1—C1	2.945 (3)	C3—C7X	1.663 (8)
La1—C2	2.965 (2)	C4—C3^i^	1.366 (5)
La1—C3	2.957 (3)	C4—C8	1.514 (6)
La1—C4	2.941 (4)	C5—H5A	0.9800
La1—Cl3	2.9128 (5)	C5—H5B	0.9800
La1—Cl4	2.9298 (6)	C5—H5C	0.9800
La1—Cl5	2.9083 (7)	C8—H8A	0.9800
La1—Cl6	2.9097 (7)	C8—H8B	0.9800
La1—Cg1	2.613 (3)	C8—H8C	0.9800
Cg1—Cg2	3.612 (4)	C6—H6A	0.9800
La1—Cl3^i^	2.9128 (5)	C6—H6B	0.9800
La1—Cl4^i^	2.9298 (6)	C6—H6C	0.9800
La1—C3^i^	2.957 (3)	C7—H7A	0.9800
La1—C2^i^	2.965 (2)	C7—H7B	0.9800
Al1—Cl1	2.0902 (10)	C7—H7C	0.9800
Al1—Cl2	2.0918 (10)	C6X—H6X1	0.9800
Al1—Cl3	2.1828 (9)	C6X—H6X2	0.9800
Al1—Cl4	2.1855 (10)	C6X—H6X3	0.9800
Al2—Cl7	2.0937 (9)	C7X—H7X1	0.9800
Al2—Cl7^i^	2.0937 (9)	C7X—H7X2	0.9800
Al2—Cl6	2.1936 (12)	C7X—H7X3	0.9800
Al2—Cl5	2.1987 (12)	C9—C10	1.389 (5)
C1—C2^i^	1.387 (4)	C9—C10^i^	1.389 (5)
C1—C2	1.387 (4)	C9—H9	0.9500
C1—C5	1.513 (6)	C10—C11	1.343 (5)
C2—C3	1.389 (5)	C10—H10	0.9500
C2—C6	1.505 (10)	C11—C12	1.344 (4)
C2—C6X	1.635 (10)	C11—H11	0.9500
C3—C4	1.366 (5)	C12—C11^i^	1.344 (4)
C3—C7	1.525 (7)	C12—H12	0.9500
			
Cl5—La1—Cl6	71.09 (2)	Cl7—Al2—Cl5	108.96 (4)
Cl5—La1—Cl3	136.497 (15)	Cl7^i^—Al2—Cl5	108.96 (4)
Cl6—La1—Cl3	82.586 (17)	Cl6—Al2—Cl5	100.72 (5)
Cl5—La1—Cl3^i^	136.497 (14)	Al1—Cl3—La1	96.16 (3)
Cl6—La1—Cl3^i^	82.586 (17)	Al1—Cl4—La1	95.61 (3)
Cl3—La1—Cl3^i^	69.61 (2)	Al2—Cl5—La1	94.06 (4)
Cl5—La1—Cl4	71.868 (13)	Al2—Cl6—La1	94.13 (4)
Cl6—La1—Cl4	76.576 (13)	C2^i^—C1—C2	119.9 (4)
Cl3—La1—Cl4	68.636 (17)	C2^i^—C1—C5	119.99 (18)
Cl3^i^—La1—Cl4	135.147 (17)	C2—C1—C5	119.99 (18)
Cl5—La1—Cl4^i^	71.868 (13)	C2^i^—C1—La1	77.23 (17)
Cl6—La1—Cl4^i^	76.576 (13)	C2—C1—La1	77.23 (17)
Cl3—La1—Cl4^i^	135.147 (17)	C5—C1—La1	119.9 (3)
Cl3^i^—La1—Cl4^i^	68.636 (17)	C1—C2—C3	119.5 (3)
Cl4—La1—Cl4^i^	140.15 (3)	C1—C2—C6	136.6 (6)
Cl5—La1—C4	74.37 (8)	C3—C2—C6	103.8 (6)
Cl6—La1—C4	145.46 (8)	C1—C2—La1	75.63 (16)
Cl3—La1—C4	124.47 (6)	C3—C2—La1	76.11 (16)
Cl3^i^—La1—C4	124.47 (6)	C6—C2—La1	120.4 (4)
Cl4—La1—C4	92.86 (3)	C6X—C2—La1	123.2 (4)
Cl4^i^—La1—C4	92.86 (3)	C4—C3—C2	120.1 (3)
Cl5—La1—C1	130.25 (7)	C4—C3—C7	98.4 (6)
Cl6—La1—C1	158.66 (7)	C2—C3—C7	141.3 (6)
Cl3—La1—C1	79.93 (6)	C4—C3—La1	76.0 (2)
Cl3^i^—La1—C1	79.93 (6)	C2—C3—La1	76.76 (15)
Cl4—La1—C1	107.879 (16)	C7—C3—La1	118.9 (3)
Cl4^i^—La1—C1	107.879 (17)	C3^i^—C4—C3	120.8 (4)
C4—La1—C1	55.88 (10)	C3^i^—C4—C8	119.5 (2)
Cl5—La1—C3^i^	87.49 (8)	C3—C4—C8	119.5 (2)
Cl6—La1—C3^i^	148.34 (6)	C3^i^—C4—La1	77.2 (2)
Cl3—La1—C3^i^	127.81 (6)	C3—C4—La1	77.2 (2)
Cl3^i^—La1—C3^i^	98.90 (10)	C8—C4—La1	121.9 (3)
Cl4—La1—C3^i^	119.39 (10)	C1—C5—H5A	109.5
Cl4^i^—La1—C3^i^	74.68 (7)	C1—C5—H5B	109.5
C4—La1—C3^i^	26.77 (10)	H5A—C5—H5B	109.5
C1—La1—C3^i^	47.94 (8)	C1—C5—H5C	109.5
Cl5—La1—C3	87.49 (8)	H5A—C5—H5C	109.5
Cl6—La1—C3	148.34 (6)	H5B—C5—H5C	109.5
Cl3—La1—C3	98.90 (10)	C4—C8—H8A	109.5
Cl3^i^—La1—C3	127.81 (6)	C4—C8—H8B	109.5
Cl4—La1—C3	74.68 (7)	H8A—C8—H8B	109.5
Cl4^i^—La1—C3	119.39 (10)	C4—C8—H8C	109.5
C4—La1—C3	26.77 (10)	H8A—C8—H8C	109.5
C1—La1—C3	47.94 (8)	H8B—C8—H8C	109.5
C3^i^—La1—C3	47.35 (17)	C2—C6—H6A	109.5
Cl5—La1—C2^i^	114.49 (8)	C2—C6—H6B	109.5
Cl6—La1—C2^i^	154.88 (5)	C2—C6—H6C	109.5
Cl3—La1—C2^i^	104.12 (8)	C3—C7—H7A	109.5
Cl3^i^—La1—C2^i^	77.41 (6)	C3—C7—H7B	109.5
Cl4—La1—C2^i^	128.51 (5)	C3—C7—H7C	109.5
Cl4^i^—La1—C2^i^	82.04 (6)	C2—C6X—H6X1	109.5
C4—La1—C2^i^	47.68 (9)	C2—C6X—H6X2	109.5
C1—La1—C2^i^	27.14 (7)	H6X1—C6X—H6X2	109.5
C3^i^—La1—C2^i^	27.13 (10)	C2—C6X—H6X3	109.5
C3—La1—C2^i^	55.61 (8)	H6X1—C6X—H6X3	109.5
Cl5—La1—C2	114.49 (8)	H6X2—C6X—H6X3	109.5
Cl6—La1—C2	154.88 (5)	C3—C7X—H7X1	109.5
Cl3—La1—C2	77.41 (6)	C3—C7X—H7X2	109.5
Cl3^i^—La1—C2	104.12 (8)	H7X1—C7X—H7X2	109.5
Cl4—La1—C2	82.04 (6)	C3—C7X—H7X3	109.5
Cl4^i^—La1—C2	128.51 (5)	H7X1—C7X—H7X3	109.5
C4—La1—C2	47.68 (9)	H7X2—C7X—H7X3	109.5
C1—La1—C2	27.14 (7)	C10—C9—C10^i^	119.1 (5)
C3^i^—La1—C2	55.61 (8)	C10—C9—H9	120.5
C3—La1—C2	27.13 (10)	C10^i^—C9—H9	120.5
C2^i^—La1—C2	47.76 (11)	C11—C10—C9	119.2 (4)
Cl1—Al1—Cl2	115.58 (4)	C11—C10—H10	120.4
Cl1—Al1—Cl3	110.99 (4)	C9—C10—H10	120.4
Cl2—Al1—Cl3	111.24 (4)	C10—C11—C12	121.1 (4)
Cl1—Al1—Cl4	110.26 (4)	C10—C11—H11	119.5
Cl2—Al1—Cl4	109.45 (5)	C12—C11—H11	119.5
Cl3—Al1—Cl4	97.89 (3)	C11^i^—C12—C11	120.5 (5)
Cl7—Al2—Cl7^i^	116.10 (6)	C11^i^—C12—H12	119.8
Cl7—Al2—Cl6	110.49 (4)	C11—C12—H12	119.8
Cl7^i^—Al2—Cl6	110.49 (4)		

Symmetry codes: (i) *x*, −*y*+1/2, *z*.
